# Current status and factors of periodontal disease among Japanese high school students: a cross-sectional study

**DOI:** 10.1038/s41405-023-00149-5

**Published:** 2023-07-14

**Authors:** Satoru Haresaku, Akiko Chishaki, Junko Hatakeyama, Yasunori Yoshinaga, Junko Yoshizumi, Mito Yamamoto, Etsuko Matsuzaki, Ippei Hamanaka, Takashi TsutsumI, Yusuke Taniguchi, Kimiko Ohgi, Masahiro Yoneda

**Affiliations:** 1 Department of Nursing, Fukuoka Nursing College, 2-15-1 Tamura, Sawara-ku, Fukuoka, 814-0193 Japan; 2 Medical Examination Center, Fukuoka Dental College, Medical and Dental General Hospital, 2-15-1 Tamura, Sawara-ku, Fukuoka, 814-0193 Japan; 3 Section of General Dentistry, Department of General Dentistry, Fukuoka Dental College, 2-15-1 Tamura, Sawara-ku, Fukuoka, 814-0193 Japan; 4 Section of Periodontology, Department of Odontology, Fukuoka Dental College, 2-15-1 Tamura, Sawara-ku, Fukuoka, 814-0193 Japan; 5 Oral Medicine Research Center, Fukuoka Dental College, 2-15-1 Tamura, Sawara-ku, Fukuoka, 814-0193 Japan; 6 Section of Oral Oncology, Department of Oral and Maxillofacial Surgery, Fukuoka Dental College, Tamura, Sawara-ku, Fukuoka, 814-0193 Japan; 7 Fukuoka Dental Hygienist School, 1-12-43 Daimyo, Chuo-ku, Fukuoka, 810-0041 Japan; 8 Section of Operative Dentistry and Endodontology, Department of Odontology, Fukuoka Dental College, 2-15-1 Tamura, Sawara-ku, Fukuoka, 814-0193 Japan; 9 Section of Removable Prosthodontics, Department of Oral Rehabilitation, Fukuoka Dental College, 2-15-1 Tamura, Sawara-ku, Fukuoka, 814-0193 Japan; 10 The Center for Visiting Dental Service, Department of General Dentistry, Fukuoka Dental College, 2-15-1 Tamura, Sawara-ku, Fukuoka, 814-0193 Japan; 11 Section of Oral Implantology, Department of Oral Rehabilitation, Fukuoka Dental College, 2-15-1 Tamura, Sawara-ku, Fukuoka, 814-0193 Japan

**Keywords:** Dental public health, Periodontitis

## Abstract

**Introduction:**

This study aimed to investigate the prevalence of periodontal disease and the factors of the disease among high school students.

**Method:**

The participants were all students aged 15–18 years (*n* = 1202) at a high school in Japan. The data on oral health perceptions and behaviours were collected by a questionnaire survey. The prevalence of periodontal disease among them was investigated with the partial community periodontal index (PCPI). A logistic regression analysis was used to identify the factors associated with the PCPI.

**Results:**

A total of 1069 students (88.9%) participated in this study. The prevalence of gingival bleeding, calculus, pocket depth of 4–5 mm, and pocket depth of 6 mm or more were 44.2%, 42.2%, 11.4%, and 1.6%, respectively. Approximately one-third of the students had a fear of dental treatment, and only 28.4% used dental floss. The results of logistic regression analysis, adjusted for sex and school year, showed that not visiting dentists regularly, not using dental floss, brushing teeth for less than 5 min, fear of dental treatment, and drinking sports drinks frequently were positively associated with periodontal conditions.

**Conclusion:**

This study identified a high prevalence of periodontal disease among Japanese high school students aged 15–18 years and its risk factors, such as poor oral health behaviours and fear of dental treatment.

## Introduction

Periodontal disease, one of the most common oral diseases, is an oral health problem worldwide. Severe periodontal diseases are estimated to affect ~19% of the global adult population, representing more than 1 billion cases worldwide [1]. Periodontal disease is the main cause of tooth loss [2], and the disease may cause adverse neonatal outcomes [3] and systematic diseases such as cardiovascular disease [4] and diabetes [5].

Gingivitis is a mild form of periodontal disease [6, 7] and has a high prevalence of 50–90% in most populations [8]. Gingival bleeding is the most easily identifiable sign of gingivitis [9] and is reported to be associated with systemic diseases such as titis media/externa and asthma in adolescence [10]. The presence of dental calculus is also associated with the prevalence of gingival inflammation among adolescents and young adults [11] because calculus provides an optimal breeding environment for bacterial biofilm growth and triggers an inflammatory response in the surrounding gingival tissue [12]. Furthermore, persistent gingival inflammation in young adulthood is significantly associated with periodontitis and tooth loss during adulthood [13]. Therefore, it is thought that identifying the status of periodontal conditions and associated factors among adolescents is important for preventing gingivitis in adolescence and periodontitis and tooth loss in future adulthood.

A Japanese national survey of dental disease in 2016 reported that the prevalence of gingivitis (≥4 mm pocket depth) among young people aged 15–24 was 17.6% [14]. The prevalence of periodontal disease increased with age, and the prevalence was more than 50% at age 50. In addition, the prevalence increased in all generations compared to a previous survey in 2011 [14].

The partial community periodontal index (PCPI), which was developed by Ainamo et al. [15], includes examinations of periodontal conditions, gingival bleeding, dental calculus, and pocket depth in the anterior and posterior teeth. It has been used in epidemiological surveys for the disease among adolescents in many countries [16–23]. Some studies have reported associations between periodontal disease and oral health behaviours among adolescents [17, 19, 23]; however, few studies have investigated adolescents’ periodontal conditions with the PCPI and the associated factors in Japan [24].

Therefore, this study aimed to identify the prevalence of periodontal disease and the factors of the disease among high school students.

## Materials and methods

### Study population

This study was a cross-sectional study conducted in accordance with the ethical standards outlined in the 1964 Declaration of Helsinki and received ethical approval from the Ethics Committee of Fukuoka Gakuen, Fukuoka, Japan (approval no. 548). Written informed consent was obtained from all participants prior to their participation in this study.

The participants comprised first- to third-year students aged 15–18 years (*n* = 1202) in a private high school in Fukuoka city, Kyushu Island, southwestern Japan. The school had a contract with the Medical Examination Center, Fukuoka Dental College Medical and Dental General Hospital to conduct research and oral health activities in 2022. The population of Fukuoka city was ~1.63 million in 2022, and it is the fifth most populous city in Japan. A questionnaire survey and periodontal examinations were conducted among the participants to collect the data.

The questionnaires were distributed to the participants by schoolteachers in October 2022. The consent form was the first page of the questionnaire. The students were informed about the research by the schoolteachers, asked to sign the consent form if they agreed to participate, and brought the questionnaire to their periodontal examination. The periodontal examination was conducted on 17 November 2022 in the school hall. All data were collected on the day of the examination.

### Clinical examination

A periodontal examination was conducted by ten dentists using a WHO probe and a dental mirror with LED light (BSA Sakurai Ltd.). The PCPI was used to examine the periodontal condition (gingival bleeding, calculus, and pocket depth) of six permanent teeth (16, 11, 26, 36, 31, 46) based on probing pocket depth at six sites around the teeth. The second molars that were examined in a standard PCPI were excluded from this study because there were some cases in which the second molars had not erupted.

Before the study, theoretical and practical calibration training of the examiners by examining 20 dental hygienists using the PCPI under the supervision of a clinically experienced dentist (Y.Y.) and epidemiologically experienced dentist (S.H.) was conducted.

The prevalence of gingival bleeding, calculus, pocket depth 4–5 mm, and 6 mm or more were calculated. If there was even one finding at six sites around the teeth, they were counted as having the findings. Their prevalence in only posterior regions, which are not evaluated in normal school dental examinations [14], was calculated. Because the treatment needs are different in each periodontal condition, the presence of gingival bleeding, calculus, and pocket depth 4 mm or more were used as the dependent variables. With the periodontal conditions, it was replaced with “1”, and without them, it was replaced with “0”.

### Questionnaire

The questionnaire was based on a previously developed questionnaire that is normally used in the Japan Academy of Dental Human Dock [25].

The questionnaire consisted of four parts: characteristics, subjective oral symptoms, oral health perceptions, and oral health behaviours. The characteristics were sex and school year. School year, not age, was used as a variable because Japanese high school students have educational programmes and school activities with same-year students. First-year students are aged 15–16, second-year students are aged 16–17, and third-year students are aged 17–18. As independent variables, female and first-year students were replaced with “0”, male and second-year students were replaced with “1”, and third-year students were replaced with “2”.

The question items of subjective oral symptoms (4 items), perceptions of oral health (5 items), and oral health behaviours (9 items) were extracted from the previously developed questionnaire [25] and limited to items that seemed to be related to periodontal disease. The answer choices of the subjective oral symptoms and the perceptions of oral health were all “Yes” or “No”. Regarding oral health behaviours, the answer choices were “3 times or more” and “2 times or less” for the question of how many times the teeth were brushed per day, “5 min or more” and “less than 5 min” for the question of how long the students brushed for, and “Yes” and “No” for the other questions. As independent variables, the answer choices of “Yes”, “3 times or more”, and “5 min or more” were replaced with “1”, and “No”, “2 times or less”, and “less than 5 min” were replaced with “0”.

Cronbach’s alpha value was used to assess the reliability of the questionnaire. The Cronbach’s alpha values of the questionnaire in the domains of subjective oral symptoms, oral health perceptions, and oral health behaviours were 0.601, 0.519, and 0.510, respectively, and were not high [26]; however, these question items were used to investigate the associations of periodontal conditions and factors.

### Statistical analyses

A chi-squared test was used to compare differences in periodontal condition, subjective oral symptoms, oral health perceptions, and oral health behaviours between sexes and to compare differences in the prevalence of periodontal conditions in subjective oral symptoms, oral health perceptions, and oral health behaviours.

A logistic regression analysis was used to determine the associations adjusted for age and school year for each periodontal condition with characteristics, oral health perceptions, and oral health behaviours, adjusted for sex and school year.

Data were analysed with 5% significance. The statistical analyses were performed using the IBM SPSS Statistics software programme (Version 21.0; IBM Corporation, Armonk, NY, USA).

## Results

A total of 1069 students (522 males and 547 females) participated in this study. The participation rate was 88.9%.

Table 1 shows the distributions of school year, periodontal conditions, subjective oral symptoms, oral health perceptions, and oral health behaviours according to sex. The prevalence of gingival bleeding, calculus, pocket depth of 4–5 mm, and pocket depth of 6 mm or more among the students were 44.2%, 42.2%, 11.4%, and 1.6%, respectively. The prevalence in the only regions limited to the posterior region was 26.4%, 3.1%, 10.5%, and 1.4%, respectively. The percentages of having calculus and a pocket depth of 4–5 mm, having oral health perceptions regarding periodontal disease, brushing teeth for less than 5 min, not using dental floss, eating many sweet foods, and drinking sports drinks frequently in the male group were higher than in the female group (*p* < 0.05).

**Table 1:** Distribution of periodontal conditions, subjective symptoms, oral health perceptions, and oral health behaviours according to sex.

Variables	Total	Male	Female	*p* value^a^
	*n* (%)	*n* (%)	*n* (%)	
School year				
First year (15–16 years old)	442(41.3)	231(44.3)	211(38.6)	0.029
Second year (16–17 years old)	298(27.9)	150(28.7)	148(27.1)	
Third year (17–18 years old)	329(30.8)	141(27.0)	188(34.4)	
Periodontal conditions				
Gingival bleeding	473(44.2)	244(46.7)	229(41.9)	0.108
Calculus	451(42.2)	255(48.9)	196(35.8)	0.000
Pocket depth of 4–5 mm	122(11.4)	70(13.4)	52(9.5)	0.045
Pocket depth of 6 mm or more	17(1.6)	8(1.5)	9(1.6)	0.883
Periodontal conditions limited only to posterior regions				
Gingival bleeding	282(26.4)	128(24.5)	154(28.2)	0.178
Calculus	33(3.1)	20(3.8)	13(2.4)	0.169
Pocket depth of 4–5 mm	112(10.5)	66(12.6)	46(8.4)	0.024
Pocket depth of 6 mm or more	15(1.4)	7(1.3)	8(1.5)	0.866
Subjective oral symptoms				
Toothache	117(11.0)	53(10.2)	64(11.7)	0.418
Gum bleeding	206(19.3)	105(20.1)	101(18.5)	0.513
Gum swelling	93(8.7)	40(7.7)	53(9.7)	0.233
Bad breath	265(24.9)	133(25.6)	132(24.2)	0.597
Oral health perceptions				
Knowledge of the effect of oral diseases on systematic diseases	683(64.0)	310(59.5)	373(68.2)	0.003
Knowledge of periodontal disease	569(53.3)	255(48.9)	314(57.5)	0.005
Interest in oral health	419(39.2)	199(38.2)	220(40.2)	0.498
Fear of dental treatments	350(32.8)	164(31.4)	186(34.1)	0.346
Knowledge of how many teeth they have	91(8.5)	37(7.1)	54(9.9)	0.103
Oral health behaviours				
Frequency of toothbrushing per day (≧3 times)	124(11.6)	49(9.4)	75(13.7)	0.027
Time for toothbrushing per brushing (≧5 min)	529(49.5)	269(51.5)	260(47.5)	0.191
Toothpaste use	835(78.3)	402(77.2)	433(79.3)	0.396
Fluoride toothpaste use	542(50.8)	257(49.3)	285(52.2)	0.349
Dental floss use	303(28.4)	126(24.2)	177(32.4)	0.003
Eating many sweet foods	717(67.2)	317(60.8)	400(73.3)	0.000
Drinking sports drinks frequently	142(13.3)	106(20.3)	36(6.6)	0.000
Experience with toothbrushing instruction	789(73.9)	361(69.3)	428(78.2)	0.001
Visiting dentists regularly (more than once a year)	189(17.7)	85(16.3)	104(19.1)	1.000

^a^Chi-squared test.

Table 2 shows the results of univariate analysis of the associations of the prevalence of periodontal conditions with characteristics, subjective oral symptoms, oral health perceptions, and oral health behaviours.

**Table 2:** Prevalence of periodontal conditions according to characteristics, subjective oral symptoms, oral health perceptions, and oral health behaviours.

Variables	Answer choices	Gingival bleeding		Calculus		Pocket depth ≧4 mm	
Characteristics		*n* (%)	*p* value^a^	*n* (%)	*p* value^a^	*n* (%)	*p* value^a^
School year	First year	180(40.7)	0.131	172(38.9)	0.164	41(9.3)	0.136
	Second year	136(45.6)		136(45.6)		40(13.4)	
	Third year	157(47.7)		143(43.5)		43(13.1)	
Sex	Female	229(41.9)	0.108	196(35.8)	0.000	53(9.7)	0.046
	Male	244(46.7)		255(48.9)		71(13.6)	
Subjective oral symptoms							
Toothache	Yes	58(49.6)	0.210	43(36.8)	0.201	12(10.3)	0.625
	No	413(43.5)		408(42.9)		112(11.8)	
Gum bleeding	Yes	100(48.5)	0.175	88(42.7)	0.836	25(12.1)	0.798
	No	373(43.3)		361(41.9)		99(11.5)	
Gum swelling	Yes	40(43.0)	0.789	40(43)	0.864	11(11.8)	0.948
	No	433(44.5)		410(42.1)		113(11.6)	
Bad breath	Yes	118(44.5)	0.868	121(45.7)	0.190	29(10.9)	0.687
	No	352(43.9)		329(41.1)		95(11.9)	
Oral health perceptions							
Fear of dental treatments	Yes	165(47.1)	0.196	169(48.3)	0.005	40(11.4)	0.891
	No	308(43.0)		282(39.3)		84(11.7)	
Interest in oral health	Yes	190(45.3)	0.543	165(39.4)	0.130	51(12.2)	0.645
	No	282(43.5)		286(44.1)		73(11.2)	
Knowledge of how many teeth they have	Yes	36(39.6)	0.347	30(33.0)	0.063	9(9.9)	0.594
	No	437(44.7)		421(43.0)		115(11.8)	
Knowledge of the effect of oral diseases on systematic diseases	Yes	301(44.1)	0.848	282(41.3)	0.407	79(11.6)	0.952
	No	172(44.7)		169(43.9)		45(11.7)	
Knowledge of periodontal disease	Yes	260(45.7)	0.292	246(43.2)	0.437	63(11.1)	0.558
	No	212(42.5)		204(40.9)		61(12.2)	
Oral health behaviours							
Frequency of toothbrushing per day	≧3 times	46(37.1)	0.088	49(39.5)	0.522	14(11.3)	0.909
	<3 times	427(45.2)		402(42.5)		110(11.6)	
Toothbrushing time per brushing	≧5 min	222(42.0)	0.137	205(38.8)	0.024	56(10.6)	0.306
	<5 min	251(46.5)		246(45.6)		68(12.6)	
Toothpaste use	Yes	360(43.1)	0.161	354(42.4)	0.782	99(11.9)	0.650
	No	112(48.3)		96(41.4)		25(10.8)	
Fluoride toothpaste use	Yes	232(42.8)	0.339	219(40.4)	0.211	68(12.5)	0.338
	No	240(45.7)		232(44.2)		56(10.7)	
Dental floss use	Yes	125(41.3)	0.225	104(34.3)	0.001	24(7.9)	0.017
	No	346(45.3)		347(45.5)		100(13.1)	
Eating many sweet foods	Yes	325(45.3)	0.348	286(39.9)	0.024	76(10.6)	0.136
	No	148(42.3)		165(47.1)		48(13.7)	
Drinking sports drinks frequently	Yes	71(50.0)	0.141	72(50.7)	0.028	25(17.6)	0.017
	No	402(43.4)		379(40.9)		99(10.7)	
Experience with toothbrushing instruction	Yes	337(42.7)	0.081	320(40.6)	0.063	90(11.4)	0.727
	No	136(48.7)		131(47.0)		34(12.2)	
Visiting dentists regularly (more than once a year)	Yes	207(40.2)	0.009	192(37.3)	0.002	50(9.7)	0.058
	No	265(48.1)		258(46.8)		74(13.4)	

^a^Chi-squared test.

The percentage of students with gingival bleeding was significantly higher in the group that visited dentists regularly than in the group that did not (*p* < 0.01). The percentages of students with calculus were significantly higher in the male group and in the group of students who had a fear of dental treatment, ate many sweet foods, and drank sports drinks frequently than in the group of students who did not (*p* < 0.05) and significantly lower in the group of students who brushed their teeth for 5 min or more and used dental floss than in the group of students who did not (*p* < 0.01). The percentages of students with a pocket depth of 4 mm or more were significantly lower in the group that used dental floss than in the group that did not use dental floss (*p* < 0.05) and higher in the group that drank sports drinks frequently than in the group that did not (*p* < 0.05).

Table 3 shows the factors associated with periodontal conditions by logistic regression analysis adjusted for sex and school year. Regular dentist visits were negatively associated with gingival bleeding. The odds ratio (OR) and 95% confidence interval (CI) were 0.75 and 0.59–0.96, respectively. Having a fear of dental treatments was positively associated with having calculus (OR, 1.47; 95% CI, 1.13–1.91). Brushing teeth for 5 min or more, using dental floss, and regular dentist visits were negatively associated with having calculus. The ORs (95% CI) were 0.74 (0.57–0.94), 0.65 (0.49–0.86), and 0.72 (0.56–0.92), respectively. Using dental floss was negatively associated with, and drinking sports drinks frequently was positively associated with having a pocket depth of 4 mm or more. The ORs (95% CI) were 0.58 (0.37–0.93) and 1.79 (1.10–2.93), respectively.

**Table 3:** Factors associated with periodontal conditions by logistic regression analysis.

Independent variable		Gingival bleeding (presence = 1, absence = 0)			Calculus (presence = 1, absence = 0)			Pocket depth ≧4 mm (presence = 1, absence = 0)		
		Adjusted^a^			Adjusted^a^			Adjusted^a^		
		OR	(95% CI)	*p* value^b^	OR	(95% CI)	*p* value^b^	OR	(95% CI)	*p* value^b^
Fear of dental treatments	Yes = 1	1.19	(0.92–1.53)	0.195	1.47	(1.13–1.91)	0.004	0.98	(0.65–1.46)	0.903
	No = 0	1^c^	–		1^c^	–		1^c^	–	
Interest in oral health	Yes = 1	1.06	(0.83–1.36)	0.648	0.81	(0.63–1.05)	0.110	1.07	(0.73–1.57)	0.728
	No = 0	1^c^	–		1^c^	–		1^c^	–	
Knowledge of how many teeth they have	Yes = 1	0.80	(0.51–1.24)	0.316	0.66	(0.42–1.05)	0.077	2.08	(0.57–7.51)	0.265
	No = 0	1^c^	–		1^c^	–		1^c^	–	
Knowledge of the effect of oral diseases on systematic diseases	Yes = 1	0.99	(0.77–1.28)	0.960	0.94	(0.73–1.22)	0.657	1.36	(0.47–3.94)	0.570
	No = 0	1^c^	–		1^c^	–		1^c^	–	
Knowledge of periodontal disease	Yes = 1	1.16	(0.91–1.48)	0.235	1.16	(0.9–1.48)	0.247	0.92	(0.63–1.35)	0.677
	No = 0	1^c^	–		1^c^	–		1^c^	–	
Frequency of toothbrushing per day	≧3 times = 0	0.73	(0.49–1.07)	0.108	0.93	(0.63–1.37)	0.718	1.00	(0.55–1.82)	0.992
	<3 times = 1	1^c^	–		1^c^	–		1^c^	–	
Toothbrushing time per brushing	≧5 min = 1	0.83	(0.65–1.05)	0.122	0.74	(0.57–0.94)	0.015	0.81	(0.55–1.18)	0.271
	<5 min = 0	1^c^	–		1^c^	–		1^c^	–	
Toothpaste use	Yes = 1	0.84	(0.63–1.13)	0.260	1.10	(0.81–1.48)	0.551	1.19	(0.74–1.9)	0.477
	No = 0	1^c^	–		1^c^	–		1^c^	–	
Fluoride toothpaste use	Yes = 1	0.90	(0.71–1.15)	0.404	0.87	(0.68–1.12)	0.281	1.23	(0.84–1.8)	0.277
	No = 0	1^c^	–		1^c^	–		1^c^	–	
Dental floss use	Yes = 1	0.86	(0.65–1.12)	0.261	0.65	(0.49–0.86)	0.003	0.58	(0.37–0.93)	0.025
	No = 0	1^c^	–		1^c^	–		1^c^	–	
Eating many sweet foods	Yes = 1	1.18	(0.91–1.53)	0.225	0.80	(0.62–1.04)	0.095	0.79	(0.53–1.16)	0.232
	No = 0	1^c^	–		1^c^	–		1^c^	–	
Drinking sports drinks frequently	Yes = 1	1.31	(0.91–1.88)	0.149	1.35	(0.93–1.94)	0.110	1.79	(1.10–2.93)	0.020
	No = 0	1^c^	–		1^c^	–		1^c^	–	
Experience with toothbrushing instruction	Yes = 1	0.76	(0.58–1.01)	0.056	0.78	(0.59–1.04)	0.089	0.92	(0.6–1.4)	0.683
	No = 0	1^c^	–		1^c^	–		1^c^	–	
Visiting dentists regularly (more than once a year)	Yes = 1	0.75	(0.59–0.96)	0.020	0.72	(0.56–0.92)	0.009	0.73	(0.5–1.08)	0.114
	No = 0	1^c^	–		1^c^	–		1^c^	–	

*OR* odds ratio, *CI* confidence interval.

^a^Adjusted for school year and sex.

^b^Logistic regression analysis.

^c^Reference.

## Discussion

This cross-sectional study was the first to investigate the associations of the prevalence of periodontal disease with PCPI examination among Japanese adolescents with their oral health perceptions and behaviours by using both univariate and multivariable analyses. This study showed that the prevalence of gingival bleeding and calculus was more than 40%. Following the description of treatment needs by Ainamo et al. [15], the students with the periodontal conditions should be encouraged to visit dentists to receive oral hygiene instruction and remove calculus. The prevalence of a pocket depth of 4 mm or more among the students was 11.6%, and most of the pockets were limited to only posterior regions. As abnormal pocket depth seems to be caused by gingivitis or the eruption of other teeth in adolescents [27], detailed examinations by dentists are needed to diagnose them. Regular dental visits were negatively associated with calculus and abnormal pocket depth (4–5 mm) in this study, and gingivitis is reported to be a risk factor for periodontal disease [13]. Therefore, those who have an abnormal pocket depth should visit dentists for detailed examinations and continue to visit dentists regularly to prevent periodontal disease in the future.

The prevalence of calculus and pocket depth of 4 mm or more among the male group was higher than that among the female group in the present study, and this tendency was reported in previous studies in Japan [24] and other countries [16, 18–20]. In addition, the percentage of the perceptions regarding oral health and the implementation of appropriate oral health behaviour among the male group were lower than among the female group. The results suggest that lower perceptions and less appropriate behaviour might contribute to the higher prevalence of periodontal disease among males.

Less than half of the students visited dentists regularly, and the variable was significantly associated with the presence of gingival bleeding and calculus among them. Regular visits to dentists, including dental prophylaxis and toothbrushing instructions, are the main oral health behaviours for the prevention of dental disease, and their effectiveness has been established [28]. Therefore, they should continue to be encouraged to visit dentists throughout their lives for the prevention and treatment of periodontal disease. However, approximately one-third of the students had a fear of dental treatment, and the variable was associated with the presence of calculus. Fear might prevent dental visits and become a risk factor for periodontal disease. In addition, students who have a fear of dental treatment and do not visit a dentist regularly might be hesitant to visit dentists without severe dental symptoms after graduation [29].

Approximately 28% of students used a dental floss, and the variable was negatively associated with the presence of dental calculus and a pocket depth of 4 mm or more. A study in Hong Kong reported the association of periodontal disease with floss use in young adults [23]. In addition, a systematic review concluded that interdental cleaning devices were effective in reducing gingivitis and plaque [30].

Therefore, they should be educated regarding how to use it and its effectiveness in periodontal disease prevention so that they are able to use it appropriately and continue to use it throughout their lives.

Drinking sport drinks frequently was significantly associated with a pocket depth of 4 mm or more, and the results suggested that it might be a risk factor for periodontal disease among them. A previous study reported that gingivitis severity among adolescents was significantly associated with sugary drinks [31], and the mechanism was thought to be a shift in the microbiological plaque flora rather than an increase in plaque mass [32]. Moreover, a systematic review reported a significant association between free sugar-containing beverages and a higher prevalence or incidence of periodontal diseases [33]. In addition, a previous study in America suggested that clear messaging should be provided about the appropriate use of sports drinks and the potential health consequences of improper consumption [34]. Therefore, they should be educated that sports drinks contain a great deal of sugar and that drinking them frequently causes not only dental caries and obesity but also periodontal disease.

There are several limitations associated with this study. First, this study was a cross-sectional study; therefore, causal associations between periodontal conditions and the associated factors were not proven, and further longitudinal studies are needed to prove them. However, associations between the disease and some behaviours, such as visiting dentists regularly and using dental floss among adolescents, were reported in another study [23].

Regarding the reliability of the periodontal examination, ten dentists examined 1069 students with the PCPI on the same day because of school circumstances. Therefore, interexaminer errors might occur in this study. The theoretical and practical calibration training of the examiners was conducted before the study, although interexaminer kappa values were not evaluated. In addition, their working experience as a clinical dentist was more than 10 years in the same dental hospital. Moreover, the prevalence of pocket depth of 4 mm or more among adolescents aged 15–19 in the Japanese national survey with the PCPI in 2016 was 6.1% and was not significantly different from that of the present study [11].

The students of only one school that accepted the implementation of this research and oral health school activities were investigated. Therefore, further studies are needed to investigate the prevalence of periodontal disease and the factors in other communities and countries to develop standardised school health educational programmes for adolescents.

## Conclusion

This study identified a high prevalence of periodontal disease among Japanese high school students aged 15–18 years and its risk factors, such as poor oral health behaviours and fear of dental treatment. Further studies are needed to investigate the prevalence of periodontal disease among adolescents and factors in other communities and countries to develop standardised and effective programmes.

## Data Availability

The datasets generated and/or analysed during the current study are not publicly available, as ethics approval was granted on the basis that only the researchers involved in this study could access the deidentified data. The raw data have been stored securely at Fukuoka Nursing College.
